# Systems biology intertwines with single cell and AI

**DOI:** 10.1186/s12859-019-2731-7

**Published:** 2019-05-01

**Authors:** Yong Wang, Xiang-Sun Zhang, Luonan Chen

**Affiliations:** 1 CEMS, NCMIS, MDIS, Academy of Mathematics and Systems Science, Chinese Academy of Sciences, Beijing, 100190 China; 2 School of Mathematical Sciences, University of Chinese Academy of Sciences, Beijing, 100049 China; 3 Key Laboratory of Systems Biology, SIBS-Novo Nordisk Translational Research Centre for PreDiabetes, Shanghai Institutes for Biological Sciences, Chinese Academy of Sciences, Shanghai, 200031 China; 4 Center for Excellence in Animal Evolution and Genetics, Chinese Academy of Sciences, Kunming, 650223 China

**Keywords:** Systems biology, Network, Data integration

## Abstract

A report of the 12th International Conference on Systems Biology (ISB2018), 18–21 August, Guiyang, China.

## Background

To study the complex genotype phenotype mapping, there are two distinct scientific paradigms. The reductionist approach decomposes the phenotypic variability step by step with the assumption that the complex life system is constructed from the additive effect of their molecules. In contrast, systems biology choses to represent the whole system by investigating the networks, which are the large scale interactions of many molecules in a systematic and simultaneously way.

The recent trend is that the two approaches are becoming increasingly complementary to each other and intertwined. The recent years witnessed the breakthrough of single cell sequencing technology, which enable to look into complex interactions at systems level inside reductive single cell level. Single cell analysis has been beyond the capabilities of ‘Omics’ technologies. This is rapidly changing with the recent examples of single cell genomics, transcriptomics, proteomics, and metabolomics.

Single cell data helps to address the cellular heterogeneity arising from stochastic expression of genes, proteins, and metabolites is a fundamental principle of cell biology. Single cell analysis is the new frontier in Omics, and single cell Omics has the potential to transform systems biology through new discoveries derived from cellular heterogeneity [1].

In addition to the single cell level, another trend is that systems biology is boosted by the artificial intelligence (AI) development. The reason is simple. In the era of big biological data, systems biology needs to mine biological insights from genome and transcriptome expression generated using next-generation sequencing and other technologies and further integrate with proteomes and metabolomes data. We noticed that big data analysis is required in various other fields such as machine learning and AI. For example, deep-learning algorithms take raw features from an extremely large, annotated data set, such as a collection of images or genomes, and use them to create a predictive tool based on patterns buried inside. These algorithms have recently shown impressive results across a variety of domains. The ideas and methods developed there can be borrowed for systems biology studies. Biology and medicine are data-rich disciplines, but the data are complex and often ill-understood. Hence, deep learning techniques may be particularly well suited to solve problems of these fields [2]. We can expect deep learning methods to provide valuable means for speeding up or aiding human investigation in future systems biology study.

Along with those new trends, this energetic interdisciplinary field has kept attracting excellent scientists and making significant progresses to convert the biological data to fundamental insights in biology and medicine. Our International Conference on Computational Systems Biology (ISB), launched 11 years ago [3–8], continues to serve as a high-quality platform and brought many researchers and students to freely exchange ideas. The 12th International Conference on Computational Systems Biology (ISB2018) was successfully co-organized by Computational Systems Biology Society of ORSC, Molecular Systems Biology Division of CSBMB, SIBS Guian Bio-Med Big Data Center, Guizhou Normal University School of Life Sciences, Guizhou Science and Technology Information Center. We hope that the joint efforts of societies, funding agencies, research institutes, and universities will further push the development of computational methodologies, algorithms, and software in systems biology.

### Meeting report

The 12th International Conference on Computational Systems Biology (ISB 2018) was held in Guiyang, China, August 18–21, 2018. The conference is supported and sponsored by Academy of Mathematics and Systems Sciences of CAS (AMSS), Shanghai Institutes for Biological Sciences of CAS (SIBS), National Natural Science Foundation of China (NSFC), Functional Genome Informatics and Systems Biology Society of CSCB, Systems Biology Technical Committee of IEEE SMC Society.

ISB2018 attracted great leading scientists working in biology, physics, mathematics and computer science, optimization, statistics, and many other mathematical methods. Following the successful ISB conferences series since 2007, ISB 2018 aims to extend the international forum for scientists, researchers, educators, and practitioners to exchange ideas and approaches, to present research findings and state-of-the-art solutions in this interdisciplinary field, including theoretical methodology development and its applications in biosciences and researches on various aspects of computational systems biology.

One hundred and five submissions to ISB2018 cover wide range of computational systems biology. Moreover, the reviewers from the Program Committee of ISB2018 selected 13 papers to be recommended for a special issue in BMC Systems Biology after significant extension of their original submission. Each submission has been peer reviewed and evaluated by three independent reviewers on the quality, originality, soundness, and significance of its contributions. Here we focus on some of the highlights of the meeting by categorizing and briefly introducing these selected papers.

Weber et al. aims to statistically infer the effective regulatory factors that are responsible for the gene expression differentiation with the help from the binding data between factors and genes. They propose a definition of binding strength based on a probability model to allow the inferences to be carried out in silico. Wang et al. propose a new generalized gene co-expression analysis algorithm via subspace clustering that can identify biologically meaningful gene co-expression modules with genes that are not all highly correlated. Hao et al. carried out an integrated analysis of lipid metabolism in cancers originating from different tissues using multiple omics data from TCGA and may provide novel insight into the understanding of tumorigenesis and progression. Hu et al. proposed a novel motif discovery algorithm based on support vector machine (MD-SVM) to learn a discriminative model for TF binding sites under a computational framework of multiple instance learning (MIL). Shih et al. proposed a voting mechanism-based (LE) prediction system to analyze any two differently clustered pathogen groups, allowing both conserved and exclusive LEs to be identified simultaneously. Liang et al. developed a single cell-based multi-scale spatio-temporal model to simulate vascular tumor growth and drug response, based on VEGFR signaling pathways, EGFR signaling pathway and cell cycle as well as several micro environmental factors that determine cell fate switches in a temporal and spatial context. Xu et al. provided a therapeutic effect recognition strategy is provided based on dynamic network biomarkers (DNB) for CML patients’ gene expression data by transforming the undirected interaction network to directed network by employing Granger causality test, followed by the predictors screened with the use of the stepwise character selection algorithm. Aouiche et al. proposed a powerful and versatile framework to identify stage-specific cancer genes and their related dynamic modules by integrating multiple datasets. Zhang et al. employed ten potential computed tomography angiography features to develop a predictive model for intracerebral haemorrhage by employing an experimental design, statistical test, and classification algorithms, which is efficient in predicting the probability of intracerebral haemorrhage. Yu et al. developed a community-driven open-source Ontology of Drug Adverse Events (ODAE) to provide a general representation of ADEs given different conditions and can be used for querying scientific questions. Luo et al. developed a strategy to screen the synergistic combinations of two drug targets in disease networks based on the classification of single drug targets. The method tried to identify the sensitivity of single intervention and then the combination of multiple interventions that can restore the disease network to a desired normal state. Chen et al. proposed a novel model-based algorithm, named VPAC, for accurate clustering of single-cell transcriptomic data through variational projection, which assumes that single-cell samples follow a Gaussian mixture distribution in a latent space.

## Abbreviations

AI: Artificial intelligence
ISB: International Conference on Computational Systems Biology
